# 

**Published:** 1856-01-01

**Authors:** 


### CONTENTS.

PAGE

A Psychological Quarterly Retrospect . . . . . . . . . . i—xxiv

PART I.—ORIGINAL COMMUNICATIONS.

1. The War . . . . . . . . . . . . . . . . . . . . 1

2. On Somnambulism . . . . . . . . . . . . . . . . . 22

3. On the Connexion between Morbid Physical and Religious Phenomena 39

4. Notes of a Visit to the Public Lunatic Asylums of Scotland . . . 51

5. Autobiography of the Insane . . . . . . . . . . . . . 66

6. On some Unrecognised Forms of Mental Disorder . . . . . . . 82

PART II.—REVIEWS.

1. The Diagnosis of Diseases of the Brain, Spinal Cord, Nerves, and their Appendages . . . . . . . . . . . . . . . . . . 90

PART III.—FOREIGN PSYCHOLOGICAL LITERATURE.

1. Medico-Legal Consultation on a Case of Monomania . . . . . . 101

2. On the Identity of Dreaming with Insanity . . . . . . . . . 102

3. Pathological condition of the Brain in Epileptics . . . . . . . 106

4. A Peculiar Form of Insanity in Children . . . . . . . . . . 107

5. On the Organic Cause of Mental Alienation, accompanied by General Paralysis . . . . . . . . . . . . . . . . . . . 109

6. Structure of the Cortical Layer of the Convolutions of the Brain . . 110

7. The American Journal of Insanity . . . . . . . . . . . . 110

PART IV.—JUDICIAL DEPARTMENT.

1. An Exposition of the Law relating to Chancery Lunatics . . . . 111

2. Important Medico-Legal Trial—The Plea of Insanity . . . . . . 126

